# Evaluation of Collaborative Robot Sustainable Integration in Manufacturing Assembly by Using Process Time Savings

**DOI:** 10.3390/ma15020611

**Published:** 2022-01-14

**Authors:** Roque Calvo, Pilar Gil

**Affiliations:** Department of Mechanical, Chemical and Industrial Design Engineering, Escuela Técnica Superior de Ingeniería y Diseño Industrial, Universidad Politécnica de Madrid, Ronda de Valencia 3, 28012 Madrid, Spain; pilar.gilsalgado@alumnos.upm.es

**Keywords:** assembly automation, cobot, automation decision support system, sustainable manufacturing, Industry 4.0, manufacturing and society

## Abstract

Collaborative robots are enablers of flexibility in the current dynamic and uncertain manufacturing environment. Decision making on its implementation requires technical feasibility, involving productivity and workforce implications that should be faced in an integrated perspective in processes where many components of different materials are assembled in products of increasing diversity and complexity. This study introduces two new parametric models for collaborative robotics, formulated in order to evaluate the differential cost of assembly (economic dimension) and the differential income from taxes that supports short-term workforce displacement (social dimension) in cobot implementation. Updated techno-economical parameters are selected for assessing feasibility ranges of application in different production scenarios. Next, the influence curves of productivity gain for a feasible implementation of cobot establish thresholds for decision making under both criteria. The results show the need for productivity gains that are significantly lower in high-wage scenarios than in low-wage scenarios; however, in a joint approach, breakeven productivity gain is always higher for the social dimension threshold than for the economic requirement of cost-effective manufacturing, with a higher gap in low-wage cases. The detailed analysis of a real case study of cobot implementation for assembly demonstrates the practical application of models and potential for future research.

## 1. Introduction and Background

Manufacturing assembly at the end of the processes of material transformation has become a key productive factor. Growing product complexity and variety require a design of components based on different materials for diverse functional purposes or only appearance; thus, the processes of joining or assembly different materials constitute a necessary final processing step of transformation for sustaining current industrial activity. Current and future manufacturing will include increasing activity towards mass customization in global markets of high competitiveness, where assembly is a key value-added activity at the end of the manufacturing supply chain. Complex and high variety products are ordinarily finished in assembly plants with different degrees of automation. After World Word II, dedicated automation of transfer lines was the paradigm of mass production. Then, technological development associated with electronics, computers and information systems promoted, first, flexible manufacturing systems and, later, the increasing implementation of robots in manufacturing tasks. The decision making of task allocation between operators and robots has been ordinary driven by physical effort and productivity in layouts with clear separation of operators from robots for security reasons. The more recent development of robots with sensors (e.g., vision, torque, force and/or contact) provides robots with the capability of safe reaction to unexpected occurrences at the workstation. This allows sharing operators’ tasks with collaborative robots, also named cobots. They are suitable for the current production trend towards mass customization, where intermediate volume batches of customized products are produced [1,2]. There is an aim at coping with uncertainty of fluctuating demand of a wide variety of customized products through smaller batches that demand building flexible assembly of quick reconfiguration, where easy programming of cobots by guidance contributes to its quick reconfiguration for multiple tasks. Collaborative robots also allow human operators to avoid repetitive and routine tasks, facilitating in placing operators’ focus on higher-level or more creative tasks. In addition, cobots can act as a smart tool, which supports handling inconvenient loads, or can overcome uncomfortable movements, reducing health problems related to ergonomics [3]. In general, cobots are used in routine tasks for applications in which the required level of precision is difficult to attain by human workers or for tasks that involve moving big loads or making uncomfortable movements. Furthermore, in order to respond to safety and security aspects of collaborative work, in 2016 technical specification ISO/TS 15066 was issued for the first time based on the already existing ISO 10218/ANSI R15.06-2012, allowing direct robot–human interaction. This standard defines five main characteristics for all cobots that are the basis of their differences with industrial robots: providing a visual indicator when the robot is operating in cooperation with a human; providing a controlled stop; allowing the operator the possibility of performing manual guidance without the need to deactivate servomotors; supervision of speed and safety distance; and the limitation of power and force by inherent design or control [4]. Collaborative robotics research can be tracked through recent reviews on the topic [5,6], laboratory studies [7,8] or the real field applications that can be found in an increasing number by examining the literature [9,10,11]. Globalization of manufacturing requires considering cobot implementation in several facets for sustainable integration. Manufacturing focus has evolved from traditional productive factors of cost, quality, time and flexibility [12] by gaining relevance in terms of its flexibility in operational and strategic dimensions [13] in order to mitigate market uncertainty and search for a sustainable balance between manufacturing performance and environmental and social impacts [14]. In general, the interaction of human workers and machines is complex with multiple aspects of interest in assembly task integration with supporting technologies for cooperation [15] and its integration in production through a sustainability framework [16]; however, this study is focused on the differential factors of implementation in assembly tasks where cobots play a genuine role in sharing tasks, which involves analyzing them as an alternative to feasible human manual assembly tasks in both the cost dimension and potential thresholds of compensation for manpower displacement.

Years ago, the degree of freedom was handled as the main driver of robot cost [17]. Recent studies on industrial robots based on one maker product line [18] found a clear correlation between payload and reach but a weaker correlation between price and payload, with no correlation with precision. In other studies, the range of payload has been identified as one of the main drivers of cobot price in the market [19] but with high variability. The cobot market is growing annually by about 50%, suggesting that the balance between technical performance and price has not been reached across its technological evolution. Cobot can find proper applications in the scenario of mass customization and in hybrid systems between full automation and manual systems, where equipment is properly organized with human resources for productivity and flexibility [20]. They are systems where flexibility has an increasing value, and it is a genuine feature of cellular systems [21] that combines a higher utilization of workforce at the price of a suboptimal use of machinery in order to respond to the uncertainty of operations from product variety and technical changeovers. Conversely, productivity with high workforce and equipment utilization is obtained from balanced flow lines that are scarcely flexible. The manufacturing system of reference for cobots in assembly is a hybrid system of operators and robots sharing assembly tasks in between full manual assembly and full automation processes [22]. The economic approach to collaborative robotics should be limited a priori to tasks that can be tackled by both robot and operator, disregarding other tasks that, due to precision, complexity or payload, are out of the bounds of effective collaboration; thus, based on their content, they are proper tasks only for operators or only for robots. The operation cost of automation has been approached in formal decision-making models in the eighties [17,23], but only full automation was modeled by including the separation of robot tasks from operators. Recently, several studies have been focused on comparing collaborative and non-collaborative robotics [24] or task allocation problem in human–robot collaboration (HRC) [25,26,27]. These models pursue the evaluation of the collaborative manufacturing process task itself but not the differential cost of alternatives based on integrated metrics and influences such as the level of productivity, equipment cost as an investment or labor cost along time. In the present research, a new parametric cost model was developed, which assesses simultaneous participation in the workplace of operators and cobots. It evaluates the overall differential cost of equipment and labour cost for decision making based on the different levels of productivity attainable in the processes by formulating an original model based on productivity gains; thus, it is applicable for evaluations for cobot implementation or for continuous improvement of the manufacturing process.

For sustainable cobot integration, the social dimension refers initially to its impact on workforce displacement. Robot implementation in 2019 reached 113 robots per 10,000 employees. In Europe, it was about 114 units; in the Americas region, it was 103 units; and in the area of Asia/Australia, it was about 118 units. Seventy-three percent of the market share is allocated in five countries: China, Japan, the United States, the Republic of Korea and Germany [28]. Figures from 2019 show a decrease with respect to the previous year of 12%, but some other reports forecast a 50% growth rate in the case of cobots [19]. The potential of routine–task automation is significant in sectors such as automotive, food, beverages, tobacco, textile and apparel and leather [29], which mostly reflects robot allocation by sectors [28]. A techno-economical reason of workforce replacement in industrial processes is the content of routine tasks, which is also promoted by the trend of automation cost decrease. From US data, in the period 1990–2010, with a base of 100% in 1990, a steady increase in salaries up to 190% approximately was reported, while a decrease in robot prices of 50% was observed in the same period [30]. In a quite overlapped period, from 1995 to 2014, together with automation increase, a decrease in the share of manufacturing in total employment of −5.1% in the US was reported but with no significant decrease in the share of manufacturing in total added value at constant prices; moreover, even an increase, when considering only goods, was observed. Therefore, an improvement in productivity, capital investment and/or methods of production seems to maintain added value with lower workforce contribution. Nevertheless, automation job replacement seems to affect lower-skill jobs associated with routine tasks [31]. In this sense, Carbonero [32] has found the impact of robotics with an emphasis on reducing offshoring from developed countries and with little significant impact in employment in developed countries, but the impact is significant in developing countries. Meanwhile, lean manufacturing has been, for years, the paradigm of manufacturing improvement techniques. It is a set of techniques based on the main participation of workers in the improvement and avoidance or elimination of waste. Without its original emphasis in automation, waste combat is well aligned with any sustainability effort. The Toyota production system has its roots in their respect towards workers and their commitment in the workplace [33]. Even when those principles were not completely adopted in Western countries, the core role of workers has continued. Currently, even with an increasing automation trend, we can find initiatives of evolving manufacturing systems around a central role of workers by Toyota [34]; thus, human-centered factories for the future are also envisioned to be compatible with Industry 4.0 development, which is sometimes referred to as the forthcoming Industry 5.0. It constitutes a remarkable strategic position for a Japanese company in a leading country in robotics and corroborates the possible integration of Industry 4.0 and lean manufacturing techniques [35]. Nevertheless, common industrial strategies around capital equipment of public companies can be based on ratios such as EBIT/value-added or added value/capital employed [36], where their product is the accounting ratio return of capital equipment (ROCE). Under this last metric, cobots seem not to be a first strategic priority because savings are partially compensated by equipment investment. Nevertheless, cobot capabilities could represent a differential manufacturing advantage despite investment criteria. In this emerging scenario of increasing robot implementation, there is a debate around the allocation of extra value created through automation and robotics in manufacturing companies. In this line, we can find suggestions with respect to regulation or taxes [29,37] that could help maintain wellbeing, facing the imbalance of low-skill workforce employment while increasing automation in some industrial sectors.

In order to assess the social impact of automation, several recent studies show its impact on employment: General studies of the potential of task automation indicate that the most automatable activities can be found in predictable physical activities that are present in the case of manufacturing task assignment. This last estimation rises from the bare point of view of task feasibility, disregarding any economic viability, regulations or social acceptance [38]. The second issue is productivity gain expectation. As reported in [32], there are two main types of recent studies from a macroeconomic point of view about the increase in productivity in economic terms and its relationship with the introduction of automation and robotics in industry. It is necessary to remark that process productivity considers time savings of production, while productivity in economic terms becomes a wider concept, sometimes measured through proxy indicators such as patent generation. In the first group of studies, in [31], a panel setting with data at the country–industry level has been used. They found weak or no economic global effects but found productivity gains. The second type of study is centered in the role of robots for local labor markets. They have found an impact effect in the US and the EU from one more robot per thousand workers: It seems to have reduced employment from 0.18 to 0.34% and wages from 0.25 to 0.50% in the US labor market (study from 1990 to 2007) [39], or according to [40] by 0.16 to 0.20% in six EU countries of their study. Other recent works [41] concluded the positive aggregated contribution of automation to progress but with impacts in labor-share displacement, forecasting a drop in labor share in value-added industry that is already shown in previous statistics.

In addition, the social dimension of employment should also consider the improvement of labor conditions at the workplace, with more complex individual effects that are very dependent on the manufacturing process. Several positive contributions have already been identified: release from tedious, repetitive or routine tasks; avoidance of overcharges on the hardest tasks in terms of effort or tasks prone to hazards due to repetition; and the improvement of the mental fatigue associated with assembly tasks (repetitive or not) [42]. Nevertheless, there are two novel aspects in the case of cobots with respect to former robot automation: They share the work with the operator at the workplace (not isolated such as common robots), and they can eventually develop enough autonomy (artificial intelligence) to replace the worker for increasing types of tasks. The first issue has been certainly approached through security standards. The second aspect can be considered inside the hard-to-assess chapter of stress induced at the job. How workers can identify with the cobot, instead of identifying machine help as a daily competitor that places their jobs at risk every day, is a matter of individual perception that is not easy to generalize and has multiple interactions [43]. The adoption of new technologies conveys these uncertainties; thus, planned adaptation could be a real conscious path for emerging technology implementation.

This study is organized as follows: In Section 2, two general parametric models of differential cost in manufacturing assembly and the main effect of work displacement supported by a short-term welfare system are developed, and joint requirements for the economic and social dimensions are compared. Each model is applied and discussed in Section 3 by using a selection of technical parameters. Next, in Section 4, a detailed analysis of a real mechanical assembly process with cobots is developed where the proposed models are applied. Finally, the concluding remarks integrate the main findings of the analysis, proposing future research lines.

## 2. Methodology

### 2.1. Differential Cost Model of Assembly with Collaborative Robots

The configuration of operator and robot-sharing tasks in a workplace can be formulated by departing from equipment cost equivalent to the cost of *N* operators per shift, working *S* shifts and *W* as current annual wages during *y* years, where *f* is the factor that establishes the equivalence of current equipment investment *C* at the average cost of capital, with savings during y years of operators’ wages, as described by Equation (1).

(1) C⋅f=N⋅S⋅y⋅W

Factor *f* can be obtained by equating the net present value of the savings of non-paid salaries during *y* years discounted at the cost of capital *r* with present investment *C*. Initial wages are *W*, with *i* being the rate of annual growth. This is the present value of an annuity [44] growing (1 + *i*) in a period of y years, as shown in Equation (2).

(2) $$C=N\cdot S\cdot \sum_{t=1}^{y}\frac{W_{t}}{{\left(1+r\right)}^{t}}=N\cdot S\cdot \sum_{t=1}^{y}\frac{W{\left(1+i\right)}^{t}}{{\left(1+r\right)}^{t}}=N\cdot S\cdot W\cdot \frac{1-{\left(\frac{1+i}{1+r}\right)}^{y}}{r-i};\;\to \frac{y}{f}=\frac{1-{\left(\frac{1+i}{1+r}\right)}^{y}}{r-i}$$

We can define the equivalent cost of equipment to replace one operator on one shift as *Q*, as described by Equation (3). The annual cost per shift of equipment that substitutes *N* operators with annual wages *W* is *N*·*W*; thus, the ratio *CW/SQ* is the annual rate per shift for equipment of initial cost *C*.

(3) $$Q=\frac{C}{N\cdot S}=\frac{y\cdot W}{f};\;\to N\cdot W=\frac{C\cdot f}{S\cdot y}=\frac{C\cdot W}{S\cdot Q}$$

In Equation (3), *W* is the current annual wages that evolve at an average rate *i* in *y* years that should include not only the direct operator substitution but also the overheads necessary to keep equipment operative (programming and maintenance).

Next, the unitary cost of an assembly of *n* parts per assembly in an assembly line with operators of *k* workstations through operator manual assembly is *C_T_*, as equated in Equation (4). It is generated from the addition of two main contributions: the operator’s rate of labor Wt ($/min), in addition to equipment cost on the same economic basis from the equivalent ratio of substitution *Q*, which is already developed in Equation (3):

(4) $$\begin{array}{l} C_{T}=t_{p}\cdot \left[W_{t}+\left(2C_{b}+N_{p}C_{c}\right)\frac{n}{k}\frac{W}{SQ}\right]=t_{p}\cdot \left[W_{t}+C_{MA}\frac{n}{k}\frac{W}{SQ}\right];\\ t_{p}=k\cdot t_{c}(1+x);\;W_{t}=\frac{n}{k}W;\;C_{MA}=2C_{b}+N_{p}C_{c} \end{array}$$

where *t_p_* is the average production time (min), *k* is the number of workstations, *t_c_* is the average time of assembly per part and *x* is the percentage of scrapped non-conforming parts for quality reasons. Concerning equipment, *C_b_* is the cost of a buffer transfer device (two are ordinarily necessary for input/output for each of the n parts); *N_p_* is the number of product changes in production that require a different conveyor or carrier *C_c_* to separate the products. The cost of the set of buffer transfer devices and conveyor carrier is named cost of equipment of manual assembly, *C_MA_*.

When a collaborative robot shares the assembly task together with an operator in a couple at the workstation, the production time becomes *t’_p_*. The new hardware includes the cost of cobot *C_cob_* and the gripper per type of product *C_g_*, and it is assumed that the flexible feeder of cost *C_ff_* provides service in the workplace to the new assignment of tasks to both the operator and the cobot. Ordinary electromechanical hardware requires a specific feeder for each product, which is also affected by the number of products and their changes. State-of-the-art technologies imply simple robots (SCARA type) with vision systems for easy reorientation and presentation (ARS Automation, 2019), which has been assumed in this analysis. Finally, the rate of labor cost is *W’*, with *W’ = mW* and with *m* > 1 counting the overheads for specific robot programming and maintenance. The aggregated cost with cobot participation is finally *CT’*, which is equated by Equation (5). The cost of a set of flexible feeders, grippers and the cobot itself is called the cost of cobot system C_CS_.

(5) $$\begin{array}{l} C_{T}{}^{\prime }=t{{}^{\prime }}_{p}\cdot \left[W_{t}+\frac{W{}^{\prime }}{SQ}\frac{n}{k}\left(C_{MA}+C_{ff}+N_{p}C_{g}+C_{cob}\right)\right]=t{{}^{\prime }}_{p}\cdot \left[W_{t}+\frac{W{}^{\prime }}{SQ}\frac{n}{k}\left(C_{MA}+C_{CS}\right)\right]\\ Where\;t{}^{\prime }{}_{p}=k\cdot t{}^{\prime }{}_{c}\cdot (1+x{}^{\prime });\;W_{t}=\frac{n}{k}\cdot W;\;W{}^{\prime }=m\cdot W;\;m>1;\;C_{CS}=C_{ff}+N_{p}C_{g}+C_{cob} \end{array}$$

The genuine collaboration of robots departs from the assumption that the task assigned to the cobot can be also performed by the operator, but its allocation in the cobot represents an advantage. The identification of suitable tasks to be assumed by the cobots represents some difficulties and a complexity factor of the study of collaborative robotics. At the beginning of automation some decades ago, the extra cost of programming and maintenance was high. Today’s ordinary spread of digital technologies in industry presumes a decreasing cost of overheads to support automation. In order to obtain a tractable model and to simultaneously approximate the reality of main influences, *m* *≈ 1* is assumed in a first-order approach. This is supported by current trends in hand guidance and programming, together with the remarkable reliability of robots [45] with consistent lifetimes of service of 8 years and low rates of breakdown [10].

We can define the percentage of time saving or productivity gain in assembly process *p* by Equation (6). It arises from a reduction in the cycle time of production *t_c_ (min)* in the assembly process with a cobot and the annual volume of production *V*, where *D* is the yearly number of working days, and *H* is the number of hours per shift.

(6) $$\begin{array}{l} p=\frac{t_{p}-t_{p}{}^{\prime }}{t_{p}}=\frac{t_{c}-t_{c}{}^{\prime }}{t_{c}};\\ V\;[\mathrm{units}\;\mathrm{year}\;\mathrm{of}\;N\;\mathrm{operators}\;\mathrm{per}\;\mathrm{shift}]=\frac{N\cdot D\cdot S\cdot H\cdot 60}{t_{p}[\min]};\;W\;[€/\min] \end{array}$$

The unitary differential cost Δ*C* of implementing cobots in the process is equated from Equations (4) and (5) in Equation (7). A similar low rate of defects *x = x’* is considered in Equation (7) for both cases; thus, a differential level of quality is initially disregarded in this first-order approach. It also allows simplifying and obtaining the explicit expression of *p* in the model.

(7) $$\Delta C=C_{T}-C_{T}{}^{\prime }=\frac{W\cdot N\cdot S\cdot D\cdot H\cdot 60}{V}\frac{n}{k}\left[p+\frac{p}{SQ}\left(C_{MA}+C_{CS}\right)-\frac{1}{SQ}C_{CS}\right]$$

In order to obtain positive unitary cost saving, the threshold or the necessary minimum productivity pmin with respect to implementing cobots is derived from Equation (7) and provided in Equation (8).

(8) $$\Delta C\geq 0\;\Rightarrow \;p_{\min}=\frac{C_{CS}}{SQ+C_{MA}+C_{CS}}$$

### 2.2. Modeling Short Term Compensation of Employment Displacement

In a first analysis of the social dimension, the direct short-term impact on low-skill employment has been clearly identified in a trade-off with productivity rise. A short- term measure is a welfare system for temporal unemployment, which provides short-term security to workers. In parallel, medium and long-term measures should involve education and training towards an evolved productive model, including cobots and other technological advances. Since the main effect on manufacturing production is manufacturing cost reduction, those differential gains would be taxed to companies; thus, society can participate in them. The model is formulated to evaluate the difference of the extra return to the society via taxes from the productivity gain with cobots during n_c_ years and the extra cost to maintain short-term welfare support during n_h_ years for displaced workers due to the increase in productivity with cobots. It is an overall approach for savings in process time as the main direct impact of overall improvement, which is described in Equation (9).

(9) $$\begin{array}{ll} \Delta S & =\left(V\cdot \Delta C-A\right)\cdot TX\cdot n_{c}-WF\cdot n_{h}\cdot N\cdot p=\\ & =\left(V\cdot \Delta C-C_{CS}\cdot \frac{N-N\cdot p}{y_{TX}}\right)\cdot TX\cdot n_{c}-WF\cdot n_{h}\cdot N\cdot p \end{array}$$

The annual volume of production *V* and product selling price are assumed constant in this analysis; thus, unitary cost savings become operative unitary revenues Δ*C*, which is previously estimated in Equation (7). Cobot system cost *C_cs_* is the differential hardware with respect to manual assembly. Amortization *A* in *yTX* years of the equipment is subtracted from the differential operating cost Δ*C* prior to tax charges at rate *TX*, as company accounting practices permit. Since production volume and time of production are proportional, for a given volume of production, there are *N*·*p* operators displaced due to productivity gain *p*. Thus, after cobot implementation for a given constant volume, only *N*(*1-p)* cobots are needed and amortized. *WF* is the annual subsidy per unemployed displaced headcount or the otherwise called net replacement income.

(10) $$\begin{array}{l} \Delta S=\left\{W\cdot N\cdot \frac{n}{k}\left[p+\frac{p}{SQ}\left(C_{MA}+C_{CS}\right)-\frac{1}{SQ}C_{CS}\right]-C_{CS}\cdot \frac{N-Np}{y_{TX}}\right\}\cdot TX\cdot n_{c}-WF\cdot n_{h}\cdot N\cdot p\\ Where\;WF,\;W\;[\$/year] \end{array}$$

By substituting Equation (7) into (9), we obtain Equation (10). This establishes the contribution of productivity through the tax rate to maintain welfare subsidy *WF* of displaced workers *N*·*p*. The operating cost of amortization has been considered, but not the extra cost of energy, which is disregarded in the first approach due to its order of magnitude in cobots [46] in comparison with capital expenditure or headcount wages. In order to consider the relationship and quantitative influences between the parameters of interest, the condition for a positive contribution or compensation, Δ*S* ≥ *0*, is deduced in (11), and the minimum productivity p_min_ is equated in Equation (12).

(11) $$\begin{array}{l} \Delta S\geq 0;\\ \left\{W\cdot N\cdot \frac{n}{k}\left[p+\frac{p}{SQ}\left(C_{MA}+C_{CS}\right)-\frac{1}{SQ}C_{CS}\right]-C_{CS}\cdot \frac{N-Np}{y_{TX}}\right\}\cdot TX\cdot n_{c}\geq WF\cdot n_{h}\cdot N\cdot p\\ p\left[\frac{n}{k}+\frac{1}{SQ}C_{MA}\frac{n}{k}+\left(\frac{1}{SQ}\frac{n}{k}+\frac{1}{y_{TX}W}\right)C_{CS}-\frac{WF\cdot n_{h}\cdot p}{W\cdot TX\cdot n_{c}}\right]\geq \left(\frac{1}{SQ}\frac{n}{k}+\frac{1}{y_{TX}W}\right)C_{CS} \end{array}$$

(12) $$\begin{array}{l} \Delta S=0;\\ p_{\min}=\frac{\left(\frac{W}{SQ}\frac{n}{k}+\frac{1}{y_{TX}}\right)C_{CS}}{W\frac{n}{k}\left(1+\frac{C_{MA}}{SQ}\right)+\left(\frac{W}{SQ}\frac{n}{k}+\frac{1}{y_{TX}}\right)C_{CS}-\frac{WF\cdot n_{h}}{TX\cdot n_{c}}}=\frac{\left(\frac{W}{SQ}\frac{n}{k}+\frac{1}{y_{TX}}\right)\frac{C_{CS}}{W}}{\frac{n}{k}\left(1+\frac{C_{MA}}{SQ}\right)+\left(\frac{W}{SQ}\frac{n}{k}+\frac{1}{y_{TX}}\right)\frac{C_{CS}}{W}-\frac{WF\cdot n_{h}}{W\cdot TX\cdot n_{c}}} \end{array}$$

### 2.3. Integrated Economic and Social Productivity Thresholds for Cobots Implementation

Former models can provide a joint analysis for decision making based on both manufacturing savings and the support of net replacement income of workforce substitution by comparing the minimum productivity thresholds of the economic dimension from Equation (8) and the social dimension from Equation (12). We wonder about which threshold is more exigent. By comparing their difference or their inverse for calculation convenience in Equation (13), it can be deduced that the productivity gain necessary to reach a contribution to a welfare system is always higher than the minimum for manufacturing economic feasibility.

(13) $$\begin{array}{ll} \frac{1}{p_{\Delta C=0}}-\frac{1}{p_{\Delta S=0}} & =\frac{p_{\Delta S=0}-p_{\Delta C=0}}{p_{\Delta C=0}\cdot p_{\Delta S=0}}=\\ & =\frac{SQ+C_{MA}}{C_{CS}}+1-\frac{\frac{n}{k}\left(1+\frac{C_{MA}}{SQ}\right)}{\left(\frac{1}{SQ}\frac{n}{k}+\frac{1}{y_{TX}W}\right)C_{CS}}-1+\frac{\frac{WF\cdot n_{h}}{W\cdot TX\cdot n_{c}}}{\left(\frac{1}{SQ}\frac{n}{k}+\frac{1}{y_{TX}W}\right)C_{CS}}=\\ & =\frac{\frac{SQ+C_{MA}}{W\cdot y_{TX}}+\frac{WF\cdot n_{h}}{W\cdot TX\cdot n_{c}}}{\left(\frac{W}{SQ}\frac{n}{k}+\frac{1}{y_{TX}}\right)\frac{C_{CS}}{W}}>0\;\Rightarrow p_{\Delta S=0}>p_{\Delta C=0} \end{array}$$

It can be observed in Equation (13) that the decreasing cost of cobot systems *Ccs* increases the gap between both thresholds, and increasing taxes *TX* reduces the difference. In addition, increasing values of the capital equivalence ratio *W/SQ* reduces that gap effectively by decreasing the numerator and increasing the denominator in Equation (13). This shows higher opportunities of implementing cobot systems in developed countries with higher salaries when seeking, in parallel, both the manufacturing cost and social contribution, despite the fact that welfare systems sustain a higher load, with higher absolute values associated with higher salaries in those countries. Meanwhile, for developing countries with lower wages, the gap between economic feasibility and the social dimension will be higher, as the model shows, despite of the fact that the threshold for economic implementation will be lower.

## 3. Results and Discussion

### 3.1. Evaluating Economic Dimension

**Figure 1:**
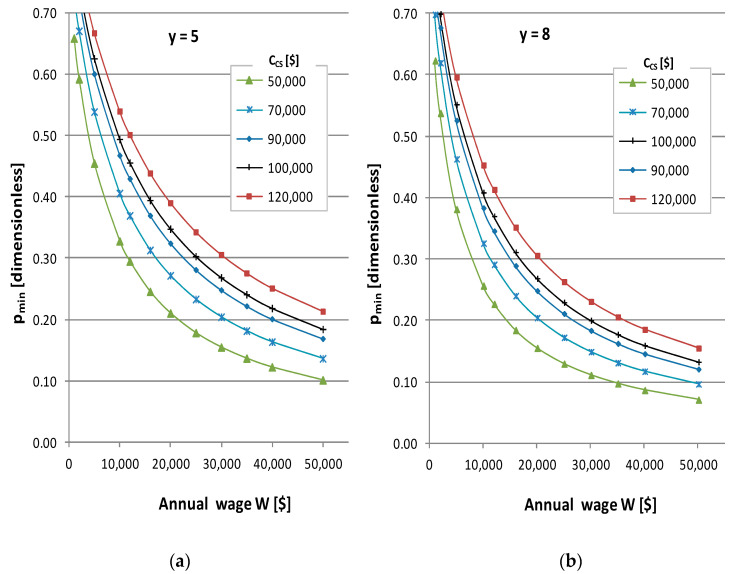
Minimum productivity pmin that supports cobot cost implementation: (**a**) y = 5; (**b**) y = 8.

According to Equation (8), increasing values of equivalent cost of equipment *Q* and the reduction in cobot system cost C_CS_ require a lower *p_min_*. In order to quantify costing trade-offs in the model (Equation (8)), the quantitative influence of different parameters is determined by using cobot cost ranges in the market from Table 1 and the selected values of Table 2 from up-to-date sources, as indicated. The influence curves from this parametrization are plotted in Figure 1.

In Figure 1a, the chart represents the breakeven point for unitary cost or minimum productivity that compensates cobot system cost implementation for an operative life of y = 5 years in a range from 50,000$ to 120,000$. Productivity gains from 15% to 30% would be necessary for a country of 30,000$ wages, but they would be from 30% to 50% if wages were only 10,000$. In Figure 1b, the effect of an extended equipment life of 8 years is represented; the necessary productivity drops to a range of 11–24% for 30,000$ wages and 25–45% for 10,000$ wages. It can be noted that, at the current technology level, an 8-year lifetime is attainable by robots, but cobot sensors and lighter construction levels, together with the risk of obsolescence, might bring accelerated shorter lifetimes ahead. Overall behavior shows that cobots implementation, from the point of view of cost, requires moderate-to-high productivity gains and is higher in low labor cost countries. Even so, in reported applications of cobots for repetitive tasks in assembly, productivity gains can reach 50% [11]; thus, in those cases integration is fully opened to a wide range of alternatives from an economic point of view, even including high-cost cobot system solutions.

### 3.2. Evaluating Social Impact

**Figure 2:**
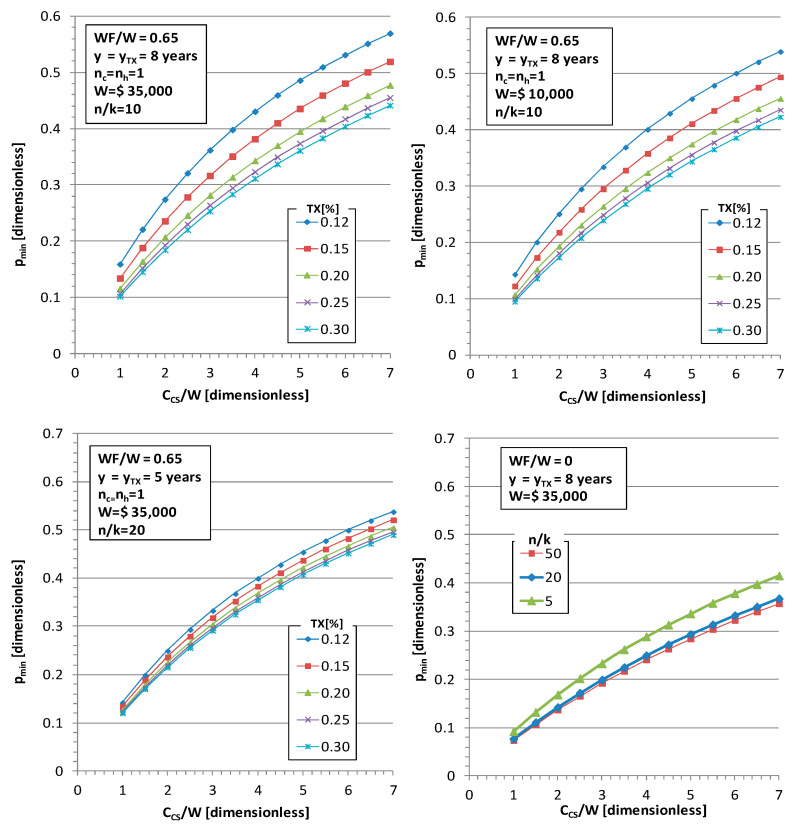
Minimum productivity pmin to support net replacement income WF.

Focusing on the assessment of quantitative influences for decision making, the parametric curves of the breakeven values of productivity are developed. In Figure 2 the parameters of Table 2 are also used for the application. One year of net replacement income for unemployment and the productivity gain necessary to support it in operations during the first year are both considered in this analysis; thus, n_c_ = n_h_ =1. In addition, accounting amortization y_TX_, cobot lifespan y of 8 years and the ratio net income replacement to wages WF/W in the first year of unemployment is in the range from 40 to 70% in numerous industrial countries [54], assuming in this initial evaluation an average of 65%. Influence curves are represented in Figure 2.

In Figure 2, top left, a cobot system (the set of flexible feeder, grippers and cobot itself) of 70,000 $ costs in a country with 35,000$ wages is observed; thus, C_CS_/W = 2, which requires 15–18% of productivity gain presenting reduced sensibility to tax levels in order to sustain 1 year of replacement income (n_h_ = 1) with 1 year of tax contribution (n_c_ = 1) at WF/W = 0.65 on the cobot system. The same hardware in a country with 10,000$ wages, C_CS_/W = 7 (Figure 2, top right), would require a productivity gain of 42–52% to sustain a similar welfare system (WF/W = 0.65) despite lower salaries or net income replacement in absolute figures. The effect of accelerated amortization because of a shorter lifetime of 5 year lows slightly alters the curves; thus, higher annual contribution via taxes is obtained. This has been combined with the effect of higher complexity assemblies with many parts per workstation (n/k), which reduces sensitivity to taxes; the approaching curves are shown in Figure 2 (bottom left).

In Figure 2 (bottom right), the case where WF = 0 is represented. In accordance with (12), it can be interpreted as the lower bound of productivity gain with some contribution to society via taxes because, under that level, productivity gain is compensated directly by amortization discount in the income statement without any positive impact in profit and loss account. The influence of n/k is low, and more complex assemblies present a lower bound than simple ones. Note, for instance, the case of a country with 35,000$ with C_CS_/W = 2; the lower bound is about *p* = 15%, but a cobot system of 120,000$ (C_CS_/W = 3.4) would require at least 20–25% productivity gains to return social contribution via taxes.

What is noteworthy is that the developed model (12) assumes a constant volume of production in order to analyze both scenarios, with and without cobot; however, in expanding markets, manufacturing productivity gained from the use of cobots could be used to increase production volumes or to gain market share by offering lower prices while retaining margins. In these other business alternatives, some extra contributions via taxes could be also obtained. A complementary and different scenario can be considered when the assembly productivity gained through robotics is directly applied to a general price reduction in goods while maintaining makers’ margins. In this case, the lower price of goods represents a direct return to society from a gain in assembly productivity, providing lower price products.

## 4. Case Study Application

Manufacturing assembly is currently one of the manufacturing activities with a higher degree of automation, particularly in electronic component assembly. Traditionally, robots with SCARA and delta configurations are used to carry out this kind of assembly, as they provide positioning precision, speed and regularity. However, very recently, collaborative robots with at least six degrees of freedom are also incorporated into assembly tasks. In addition to the former advantages, these robots can provide some flexibility that dedicated automated systems cannot offer. Delta robots can reach high speeds, but their work surface is limited and they must be mounted on the area of process operation. SCARA robots are also fast and can reach a larger area, but they are limited to four axes. The main benefit of collaborative robots is their great flexibility, with extra orientation capabilities that allow the assembly of more complicated designs that are unfeasible to automate otherwise.

**Figure 3:**
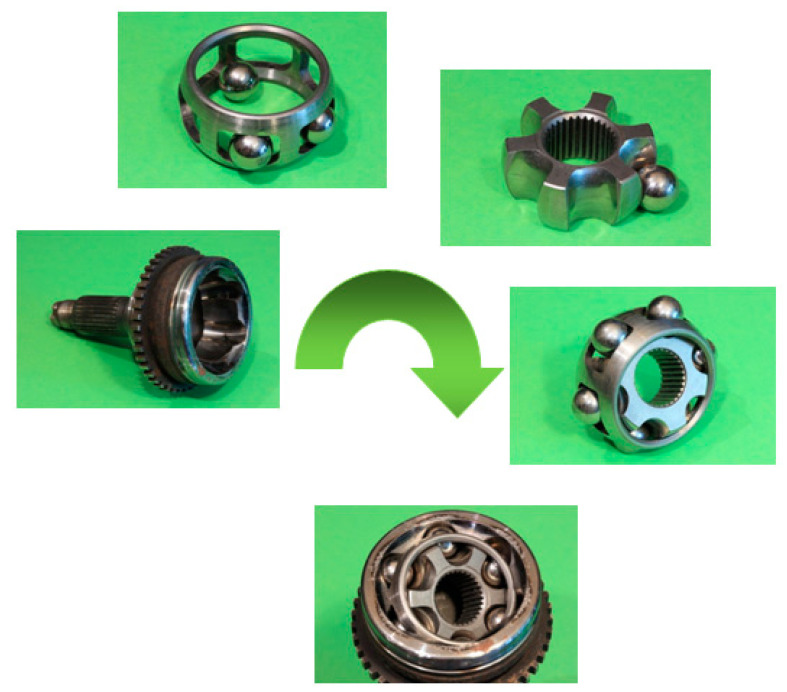
A Rzeppa homokinetic joint assembly.

In order to accomplish the application of models previously developed on a case study of collaborative assembly, the time of processing has been analyzed following the Methods-Time Measurement (MTM) standard method of predetermined times. This is the assembly of a Rzeppa homokinetic joint, also known as a ball homokinetic joint or constant velocity joint that consists of six balls housed in a cage (Figure 3). In the joint, the balls roll in a non-sliding manner with the conductive tree and with the conducted one at the same time. This coupling occurs because the balls are also housed in O-rings, which are evenly spaced along two parts inside and outside. The outer casing is attached to the drive shaft on the side of the wheel. The inner ring is the core of the drive shaft. This analysis has been developed based on the initial assembly sequence proposed in [55]. Typical material selection includes an outer ring made from medium carbon steels, such as AISI/SAE 1050, with hardened bearing surfaces; many inner ring and cage parts are made from alloy steels, such as AISI/SAE 5120 (DIN ST52-3), with carburized contact surfaces or splines. Hardening reaches from 57 to 64 HRC. Balls are typically fabricated from high carbon alloy steel, for instance SAE 5200 (EN ISO 683-17), and are hardened and tempered [56].

**Figure 4:**
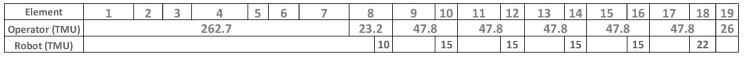
Chronogram of the Rzeppa homokinetic joint assembly.

Manual assembly is performed by using an insertion tool and includes the sequential steps shown in Table 3 and represented in the chronogram Figure 4, which are described as follows: reaching the casing and positioning the casing on the support; reaching the inner ring, reaching the cage and joining both elements by inserting the ring into the cage; reaching the balls next; positioning the cage inside the casing; inserting the first and second balls and hitting the cage with the insertion tool until it is in its correct position; inserting the tool into the inner ring hole and rotating the cage; inserting the third ball; inserting the tool into the inner ring hole and rotating the cage; inserting the fourth ball; inserting the tool into the inner ring hole and rotating the cage; inserting the fifth ball; inserting the tool into the inner ring hole and rotating the cage; inserting the sixth ball; finally removing the insertion tool and collecting the mounted gasket and placing it in a cart. The total assembly cycle time of the homokinetic joint becomes 715.8 TMU (Time Measurement Unit) or the equivalent of 25.768 s when performed manually. An experienced operator performs this operation very quickly and is able to guarantee the production of 70 homokinetic joints per hour [55]. However, the turning movements of the cage using the insertion tool can cause effort problems. From this previous ergonomic study, it was estimated that during an eight-hour shift, the operator lifts a total weight of five tons and performs eighteen thousand arm lifts. Other studies on a real assembly plant by [42] revealed that the assembly process is demanding in both physical and mental dimensions. Therefore, this activity is a good candidate to be partially automated in order to assist the operator.

**Table 3:** MTM for the assembly of a Rzeppa homokinetic joint and process timeline diagram.

Elements: Inner Ring, 6 Balls, Cage, Outer Casing. Tools: Insertion Tool Fixed to the Gripper					
Agent	Element	Action	CODE	TMU	GROUP SUM
Operator	1. Outer casing	Reach	R60C	22.3	262.7
		Grasp	G1A	2.0	
		Move	M60A	22.1	
		Position	P1SE	5.6	
	2. Inner ring	Reach	R40A	11.3	
		Grasp	G1A	2.0	
		Move	M60A	15.6	
	3. Cage	Reach	R40A	11.3	
		Grasp	G1A	2.0	
		Move	M40B	15.6	
	4. Cage and ring	Position	P3SSE	46.5	
		Apply pressure	APA	10.6	
	5. Balls	Reach	R10B	6.3	
		Grasp	G1C1	7.3	
	6. Cage and ring	Position	P2SSD	25.3	
		Turn	T45S	3.5	
	7. Ball 1	Position	P3NSD	53.4	
	8. Semi-assembled joint	Grasp	G1A	2.0	23.2
		Move	M40B	15.6	
		Position	P1SE	5.6	
Robot		Move	MA10	6.0	10.1
		Turn	T60S	4.1	
Operator	9. Ball 2	Position	P3NSE	47.8	47.8
Robot	10. Semi-assembled joint	Turn	T120S	6.8	15.0
		Turn	T60S	4.1	
		Turn	T60S	4.1	
Operator	11. Ball 3	Position	P3NSE	47.8	47.8
Robot	12. Semi-assembled joint	Turn	T120S	6.8	15.0
		Turn	T60S	4.1	
		Turn	T60S	4.1	
Operator	13. Ball 4	Position	P3NSE	47.8	47.8
Robot	14. Semi-assembled joint	Turn	T120S	6.8	15
		Turn	T60S	4.1	
		Turn	T60S	4.1	
Operator	15. Ball 5	Position	P3NSE	47.8	47.8
Robot	16. Semi-assembled joint	Turn	T120S	6.8	15.0
		Turn	T60S	4.1	
		Turn	T60S	4.1	
Operator	17. Ball 6	Position	P3NSE	47.8	47.8
Robot	18. Semi-assembled joint	Turn	T120S	6.8	22.4
		Move	M15B	15.6	
Operator	19. Semi-assembled joint	Grasp	G1A	2.0	26.1
		Move	M60A	22.1	
		Move	M2B	2.0	
TMU = time measurement unit				**TOTAL (TMU)**	**643.5**
1 h = 100,000 TMU				**TOTAL (s)**	**23.166**

In addition, inserting the balls requires great positioning precision together with adaptation skills, since the position of the cage can be variable, and any robots currently developed are unable to perform this activity satisfactorily on its own. For this reason, the proposed collaborative task assignment allows the participation of the robot along with a human operator in activities that require higher force, while the operator performs activities that require greater coordination and skills. This new joint assembly process starts with the casing pre-assembled with the cage, the inner ring and a first ball. This set is placed in the gripper of the collaborative robot. Facing the gripper, the insertion tool is placed on a fixed support. The operator guides the robot until the tool is inserted into the hole in the central ring. The robot moves the set until a gap is created between the cage and the housing and the operator inserts the second ball into this hole. The robot moves to place the ball, rotates its wrist to prepare the reception of the next ball and moves again to create the gap. The operator inserts the third ball. The above steps are repeated until all six balls have been introduced. Finally, the robot separates the mounted joint from the insertion tool, and the operator removes the mounted joint from the end of the robot. In MTM analysis, the assembly of the casing, the cage, the inner ring and the first ball is performed following the same steps as in manual assembly. Moreover, the robot gripper adopts a speed similar to that of the human operator and never exceeds the maximum speed allowed by the standards of 0.25 m/s. The total assembly cycle time of the homokinetic joint using a collaborative robot is 653.5 TMU or 23.166 s (Table 3), and the chronogram is shown in Figure 4.

Therefore, together with the additional improvements in ergonomics that are already described, assembly time involved time savings of 2.603 s or 10% in terms of productivity gain in the process, and this involved conservative cobot speeds for security precautions. This reduction in time occurs for two main reasons: Firstly, the cobot and the operator carry out movements in parallel, particularly during the sequence of collaboration tasks (elements 8 to 18); thus, the longer lead-time task determines the cycle time. This happens, for example, when the cobot rotates its wrist while the operator reaches for the next ball to be introduced. These movements were sequential in the case of manual assembly. Secondly, the cobot performs fewer movements than the human operator when carrying out its tasks. In this specific case, the cobot introduces the tool into the central ring only once and rotates the joint with respect to the tool. In fully manual human assembly, the operator had to reposition the tool several times in order to insert the balls.

Additional improvements to the process could be eventually proposed. The assembly cycle time could be reduced by dividing the assembly task into two workstations: a first station for the manual assembly of the casing, the cage, the inner ring and the first ball; and a second station for the assembly of the remaining balls with the collaborative robot so that both tasks are carried out in parallel. Even an extra reduction in cycle time could eventually be achieved by implementing a workstation that includes more than one collaborative robot so that the operator can insert balls into more than one robot at a time without having to stand still while the robot repositions the joint. Finally, the maximum speed of the gripper allowed by the ISO standard would be up to 0.25 m/s, but the speed of the cobot has been limited to being equal to and not higher than the operator’s speed in this conservative analysis. This has been assumed by considering several factors such as the stress of the worker facing a high-speed cobot or the fact that cobot programming is conducted manually [57]. However, there are some specific movements where the robot working at maximum speed could be considered an option: those of wrist turning where the cobot has a clear advantage over a human wrist by turning its gripper 360°. Not less important, this type of movement would not cause stress to the operator as they are small and very controlled movements; thus, it would be difficult for the operator to be hit by the cobot. What is noteworthy is that, in serial production, long-term assembly cycle time reduction could probably increase by taking into account the evolution of human performance in continuous duty due to fatigue and task time variability. In summary, even when more improvements could be expected, with the baseline assumption, a 10% productivity gain is initially estimated, with several extra opportunities outlined for increasing the results of improvement.

The industrial feasibility of this productivity gain can be analyzed by using the proposed models. In particular, for the parameterisation used in Figure 1, this productivity gain of a cobot system of 50,000$ with 8 years of lifetime is feasible in countries with wages over 35,000$. Nevertheless, this hardware of ratio C_CS_/W = 1.5 is under the limit of contributing, in 1 year, to net replacement income in the welfare system; thus, according to Figure 2 (upper left), minimum productivity should be 17–20%. Other more relaxed scenarios can be possible in the analysis by using the model; thus, covering the annual replacement income with taxes in one year of the displaced worker (n_c_ = n_h_ = 1) might be considered a quite demanding industrial policy. As per Figure 2 (bottom right), for a ratio of C_CS_/W = 1.5, a productivity gain of about 10–12% is the lower limit that compensates the reduction in assembly cost in the income statement by the amortization of equipment for moderate (case of study) to high ratios n/k of assembly task per workstation.

## 5. Conclusions

Two parametric models for assessing collaborative robots in manufacturing assembly processes have been developed by using relevant indicators for manufacturing cost and social impact. Process productivity gain or process time saving is used as a decision-making parameter in the evaluation of a collaborative assembly process with robots. While other models and particular studies evaluate details of the manufacturing process, once process time savings with cobots have been estimated for a particular process, the proposed parametric models can be applied for decision making with respect to different production scenarios under the sustainability dimensions of cost and social impact.

The results show that the economic justification of cobot system implementation requires moderate attainable time savings in order to become a feasible investment. There is a high non-linear influence of wages based on the equivalent cost of equipment to replace one operator on one shift, Q, that also takes into account the cost of capital and wage growth. Collaborative robots can improve productivity and replace workers in routine tasks with assembly time savings that compensate investments in short return periods in high labour cost scenarios and along longer periods in low labour cost countries. In this regard, the current implementation of cobots in low salary countries might be driven by different strategic long-term decision-making criteria instead of a return in investment. In the social dimension, collaborative robots can assume many routines together, improving general operators’ work conditions, but it can also displace workers with impacts in employment. The model shows that a social approach of worker displacement compensation through a short-term welfare system is highly determined by wages, and it can be supported more easily from taxes in high wage countries. Since equipment acquisition cost is globally quite similar, promoting cobots in low wage countries might be hardly sustainable in this social aspect, despite lower welfare compensation associated with lower salaries, as has been shown through the quantitative results. The combined requirements of manufacturing cost savings and social contribution via taxes have shown that the social threshold of productivity is higher than the economic one; thus, when trying to comply with both, the social threshold should drive decision making, particularly in low wage countries. The proposed parametric models can be updated with technology and economic evolution, and they can be integrated or become complementary to other frameworks of cobot assessment of sustainability in future studies. This allows using models for different countries or adopting the changes of technical and economic parameters that the models include due to industrial or economic evolution in a country or just in order to evaluate possible production scenarios

In a close relationship with cost, studying the real limits of collaboration is proposed for future studies between operator and robot replacement in parallel with cobot-increasing capabilities (sensors and AI); thus, it will establish useful evolving limits of task replacement beyond current estimations. Environmental impact is the third dimension of sustainability in collaborative robots that could be the subject of future study when its proliferation will make it remarkable in the context of overall industrial hardware. It is in close connection with materials and energy consumption. Current scarce field studies with respect to consumption and, specifically, materials used to construct collaborative robots fix their attention on the carbon footprint [10] by utilizing analyses of alternative metallic alloys, polymeric materials or composites used to build them. Highly processed light and high resistance materials are used in order to limit the effects of inertia; thus, the tradeoffs between performance and their lifecycle environmental impact should be assessed for proper sustainability evaluation. The study of the balance in local labor markets of robots introduction is also foreseen for future research in the social dimension, since technology access is global. Both the continuous prospect of operators’ replacement (physical or cognitive) and the improvement of working conditions should be researched in parallel in order to assess balanced evaluations. Collaborative robot technology advances in parallel in terms of increasing capacities and decreasing cost; thus, both should be subject to surveillance and research with respect to their ranges of applicability in future research.

## Figures and Tables

**Table 1:** Selected specifications and price some cobots from leading manufacturers.

Manufacturer	Model	D.O.F.	Payload (kg)	Reach (mm)	Technology	Price Approx. (k€)
KUKA	Iiwa	7	7–14	911–931	Torque sensors in all joints	50–100 [47]
Universal Robots	UR3e/5e/10e	6	3–5–10	500–850–1300	Motor current monitoring	22.7–27−35.5 [48]
Omron	TM Series	6	4–14	700–1300	Built-in vision	35–50 [49]
Doosan	M0609/1509/0617	6	6–15	900–1700	Torque sensors, electrostatic touch interface	30–45 [50]
Fanuc	CR7iA/L	6	7	717	Soft external skin, force torque sensor at the base	35 (FOB) [51]

**Table 2:** Parameter selection for the model of differential cost in assembly with collaborative robots.

Operating and Financial Parameters			
Symbol	Concept	Value	Unit
S	number of shifts	2	-
i	rate of salaries rise	2	%
r	cost of capital [52]	7	%
N_p_	number of different products assembled	10	-
C_g_	cost of cobot gripper per part	500	€
C_b_	cost of transfer device i/o per workstation [53]	5000	€
C_c_	cost of work carrier	1000	€
C_ff_	cost of a flexible parts feeder	28,000	€

## Data Availability

Not applicable.
